# Supplementary material for: Association behavior between sand tiger sharks and round scad is driven by mesopredators
Source: PeerJ. 2021 Apr 8;9:e11164. doi: 10.7717/peerj.11164 (PMC8038640; doi:10.7717/peerj.11164)
Supplement: Table S1 — Date (DD Mmm YYYYY) and time (U.S. Eastern Time, UTC/GMT -5; Eastern Standard Time (EST), Eastern Daylight Time (EDT)) are local to the camera. Time within a video is listed if the reference is to a fleeting event (H:MM:SS). Acronyms are consistent with main text, as follows: participants–STs, sand tiger shark(s), RS, round scad, MPs, mesopredator(s) of up to six species, AJ, almaco jack, BR, blue runner, CJ, crevalle jack, GA, greater amberjack, LT, little tunny; association behaviors between STs and RS (STs + RS)–NVI, no visible interaction, LA, loosely associated, TA, tightly associated. Video files are deposited in a public online repository on Zenodo (doi: 10.5281/zenodo.4477423). [file peerj-09-11164-s002.docx]

| Description | Date of occurrence | Clock time of occurrence | Video time | Video reference |
| --- | --- | --- | --- | --- |
| Study site views | See videos | -- | -- | <https://www.youtube.com/playlist?list=PLK1g13VpyT6oYUJL7U3hRPlt2U5L_mcKL> |
| RS foraging for plankton | 24 Mar 2016 | 1541 EDT | all | <https://youtu.be/7_i8hoQXeAU> |
| MPs (GA) foraging on RS, RS associating with structure | 25 Oct 2018 | 1458 EDT | 0:00:04, :13 | <https://youtu.be/IesLMb9OStw> |
| MPs (BR, LT, AJ) foraging on RS, RS associating with structure | 7 Nov 2018 | 1027 EST | all | <https://youtu.be/CTwih5UYaqw> |
| MPs (AJ) foraging on RS, RS associating with structure | 18 Jun 2016 | 1002 EDT | all | <https://youtu.be/CIFLIu2FVfA> |
| STs+RS LA | 15 Nov 2019 | 0950 EST | all | <https://youtu.be/_CIqWVUprmU> |
| STs+RS TA | 19 Dec 2015 | 1121 EST | all | <https://youtu.be/P37lg7iiDJo> |
| STs+RS TA pulsating | 18 Apr 2017 | unknown | 0:00:09 | <https://youtu.be/9WuEyByf_Pw> |
| STs+RS transition LA to TA | 22 Apr 2017 | 1057 EDT | 0:03:00, 15:25 | <https://youtu.be/1Ss-AvAMkVg> |
| STs attempted predation on LT | 19 Dec 2015 | 1121 EST | 0:00:08 | <https://youtu.be/P37lg7iiDJo> |
| STs attempted predation on LT | 06 Jan 2019 | 1040 EST | 0:00:09 | <https://youtu.be/PllHZr-ioeo> |
| STs attempted predation on red drum | 12 Jan 2019 | 1148 EST | 0:19:45 | <https://youtu.be/i5wO7ILbbd8> |
| STs attempted predation on blue runner | 12 Jan 2019 | 1150 EST | 0:20:52 | <https://youtu.be/i5wO7ILbbd8> |
| STs attempted predation on gag | 15 Jan 2019 | 0740 EST | 0:00:07 | <https://youtu.be/cfGFAq1cQtI> |
