# Supplementary material for: Association behavior between sand tiger sharks and round scad is driven by mesopredators
Source: PeerJ. 2021 Apr 8;9:e11164. doi: 10.7717/peerj.11164 (PMC8038640; doi:10.7717/peerj.11164)
Supplement: Table S2 — Bolded species are those used as MPs in analyses. References indicated with * are cited in the main text, while those indicated with parentheses and ++ appear below the table. [file peerj-09-11164-s003.docx]

| Mesopredator | Locale of observations | Source of observations | Reference |
| --- | --- | --- | --- |
| African pompano *Alectis ciliaris* | Frying Pan Tower, NC | direct in situ (SharkCam) | observation from SharkCam |
| **almaco jack *Seriola rivoliana*** | Anchor Reef, GA | direct in situ (SCUBA) | Auster et al., 2009* |
|  | South Atlantic Bight | not directly reported, inferred from unidentified teleosts in stomach contents | Manooch & Haimovici, 1983* |
|  | Frying Pan Tower, NC | direct in situ (SharkCam) | observation from SharkCam |
| **Atlantic bonito *Sarda sarda*** | Tyrrhenian Sea, Italy (Mediterranean Sea) | stomach contents contain similar fishes (eg: *Trachurus* sp. [Carangidae]) | Campo et al., 2006* |
|  | northeast Aegean Sea, Greece (Mediterranean Sea) | stomach contents contain similar fishes (eg: *Trachurus* sp. [Carangidae]) | Fletcher, Batjakas, & Pierce, 2013* |
| bar jack *Carangoides ruber* | Anchor Reef, GA | direct in situ (SCUBA) | Auster et al., 2009* |
| black sea bass *Centropristis striata* | Anchor Reef, GA | direct in situ (SCUBA) | Auster et al., 2009* |
|  | hard bottoms in Long Bay, NC | direct in situ (SCUBA) or in stomach contents (pers. obs.) | in person observation |
| **blue runner *Caranx crysos*** | Anchor Reef, GA | direct in situ (SCUBA) | Auster et al., 2009* |
|  | Frying Pan Tower, NC | direct in situ (SharkCam) | observation from SharkCam |
|  | Gulf of Gabes, Tunisia (Mediterranean Sea) | not directly reported, fed on similar prey | Sley et al., 2009* |
| bluefish *Pomatomus saltatrix* | Onslow Bay, NC, to Long Bay, SC, northwest FL, LA | stomach contents | (Naughton & Saloman, 1985a)++ |
| cobia *Rachycentron canadum* | Anchor Reef, GA | direct in situ (SCUBA) | Auster et al., 2009* |
| **crevalle jack *Caranx hippos*** | northwest FL, LA | stomach contents | Saloman & Naughton, 1984* |
| gag *Mycteroperca microlepis* | Anchor Reef, GA | direct in situ (SCUBA) | Auster et al., 2009* |
|  | hard bottoms in Long Bay, NC | direct in situ (SCUBA) or in stomach contents (pers. obs.) | in person observation |
|  | NC, northwest FL | stomach contents | (Naughton & Saloman, 1985b)++ |
|  | Frying Pan Tower, NC | direct in situ (SharkCam) | observation from SharkCam |
|  | Cape Hatteras, NC to GA | molecular identification of stomach contents | (Spanik, 2018)++ |
| great barracuda *Sphyraena barracuda* | Anchor Reef, GA | direct in situ (SCUBA) | Auster et al., 2009* |
|  | Frying Pan Tower, NC | direct in situ (SharkCam) | observation from SharkCam |
| **greater amberjack *Seriola dumerili*** | Anchor Reef, GA | direct in situ (SCUBA) | Auster et al., 2009* |
|  | South Atlantic Bight | stomach contents | Manooch & Haimovici, 1983* |
|  | Frying Pan Tower, NC | direct in situ (SharkCam) | observation from SharkCam |
| king mackerel *Scomberomorus cavalla* | Cape Hatteras, NC, to Long Bay, SC, GA, northwest FL, LA, TX | stomach contents | (Saloman & Naughton, 1983)++ |
| red lionfish *Pterois volitans* | northern Gulf of Mexico | DNA barcoding of stomach contents | (Dahl et al., 2017)++ |
|  | hard bottoms in Long Bay, NC | direct in situ (SCUBA) or in stomach contents (pers. obs.) | in person observation |
|  | Onslow Bay, NC | stable isotopes on stomach contents | (Muñoz, Currin & Whitfield, 2011)++ |
| **little tunny *Euthynnus alletteratus*** | northern Gulf of Mexico | direct in situ (snorkel) | (Hirama & Witherington, 2012)++ |
|  | NC to TX | stomach contents | (Manooch, Mason & Nelson, 1985)++ |
|  | Frying Pan Tower, NC | direct in situ (SharkCam) | observation from SharkCam |
| loggerhead sea turtle *Caretta caretta* | northern Gulf of Mexico | direct in situ (snorkel) | (Hirama & Witherington, 2012)++ |
| sandbar shark *Carcharhinus plumbeus* | Frying Pan Tower, NC | direct in situ (SharkCam) | observation from SharkCam |
| scamp *Mycteroperca phenax* | Anchor Reef, GA | direct in situ (SCUBA) | Auster et al., 2009* |
|  | hard bottoms in Long Bay, NC | direct in situ (SCUBA) or in stomach contents (pers. obs.) | in person observation |
|  | Cape Hatteras, NC to GA | stomach contents | Matheson, Huntsman, & Manooch, 1986* |
|  | Cape Hatteras, NC to GA | molecular identification of stomach contents | (Spanik, 2018)++ |
| vermilion snapper *Rhomboplites aurorubens* | hard bottoms in Long Bay, NC | direct in situ (SCUBA) or in stomach contents (pers. obs.) | in person observation |
| yellow jack *Carangoides bartholomaei* | Frying Pan Tower, NC | direct in situ (SharkCam) | observation from SharkCam |

**++References**

Dahl K, Patterson W, Robertson A, Ortmann A. 2017. DNA barcoding signiﬁcantly improves resolution of invasive lionﬁsh diet in the Northern Gulf of Mexico. *Biological Invasions* 19:1917–1933. DOI: 10.1007/s10530-017-1407-3.

Hirama S, Witherington B. 2012. A loggerhead sea turtle (*Caretta caretta*) preying on fish within a mixed-species feeding aggregation. *Chelonian Conservation and Biology* 11:261–265. DOI: 10.2744/CCB-0918a.1.

Manooch CS, Mason DL, Nelson RS. 1985. Foods of little tunny *Euthynnus alletteratus* collected along the southeastern and Gulf coasts of the United States. *Nippon Suisan Gakkaishi* 51:1207–1218. DOI: 10.2331/suisan.51.1207.

Muñoz R, Currin C, Whitfield P. 2011. Diet of invasive lionfish on hard bottom reefs of the Southeast USA: insights from stomach contents and stable isotopes. *Marine Ecology Progress Series* 432:181–193. DOI: 10.3354/meps09154.

Naughton SP, Saloman CH. 1985a. *Food of bluefish (*Pomatomus saltatrix*) from the U.S. south Atlantic and Gulf of Mexico*. NOAA Technical Memorandum NMFS-SEFC-150: U.S. Department of Commerce, National Oceanic and Atmospheric Administration, National Marine Fisheries Service, Southeast Fisheries Science Center, Panama City Laboratory, Panama City, FL.

Naughton SP, Saloman CH. 1985b. *Food of gag (*Mycteroperca microlepis*) from North Carolina and three areas of Florida*. NOAA Technical Memorandum NMFS-SEFC-160: U.S. Department of Commerce, National Oceanic and Atmospheric Administration, National Marine Fisheries Service, Southeast Fisheries Science Center, Panama City Laboratory, Panama City, FL.

Saloman CH, Naughton SP. 1983. *Food of king mackerel,* Scomberomorus cavalla*, from the southeastern United States including the Gulf of Mexico*. NOAA Technical Memorandum NMFS-SEFC-126: U.S. Department of Commerce, National Oceanic and Atmospheric Administration, National Marine Fisheries Service, Southeast Fisheries Science Center, Panama City Laboratory, Panama City, FL.

Spanik KR. 2018. Improving diet resolution for reef-associated large piscivorous predators in the U.S. southeast Atlantic using molecular tools. M. S. Thesis. Graduate School of the College of Charleston.
